# Trends, levels, and projections of Head and Neck Cancer in China between 2000 and 2021: Findings from the Global Burden of Disease 2021

**DOI:** 10.1371/journal.pone.0322533

**Published:** 2025-05-02

**Authors:** Meng Wu, Hao Chen, Wei Zhang, Xingyu Feng, Shuangyue Zhang

**Affiliations:** Department of Oral and Maxillofacial Surgery, The Affiliated Huaian No.1 People’s Hospital of Nanjing Medical University, Huaian, Jiangsu Province, China; Chung Shan Medical University, TAIWAN

## Abstract

Head and neck cancer (HNC), a condition that is both disfiguring and potentially fatal, has become a critical public health challenge. This study seeks to evaluate the trends in HNC burden and predict its future trajectory in China. Utilizing data from the 2021 Global Burden of Disease database, we focused on the incidence, mortality, and disability-adjusted life years related to lip and oral cavity, nasopharyngeal, and laryngeal cancers within the country. We analyzed changes in incidence and mortality rates using the estimated annual percentage change, age-period-cohort (APC) analysis, and decomposition analysis. Additionally, an Autoregressive Integrated Moving Average model was employed to forecast the future burden of HNC. In 2021, China’s incidence rates for lip and oral cavity, nasopharyngeal, and laryngeal cancers were higher than those in 40.98%, 98.05%, and 50.73% of countries worldwide, respectively. The burden of HNC increases significantly with age, particularly among men. The APC analysis indicates a rising incidence of HNC among younger adults. Decomposition analysis comparing 2021–2019 highlighted that ASIR and aging were the primary factors influencing the number of cases and deaths. Projections indicate that the burden of HNC in China is expected to continue rising. To combat this growing issue, it is imperative to enhance public health strategies that focus on prevention, early detection, and efficient resource allocation.

## Introduction

Head and Neck Cancer (HNC) refers to a group of aggressive tumors arising in the head and neck region, including cancers of the lip and oral cavity, pharynx, and larynx [1], with squamous cell carcinoma being the predominant type [2]. HNC frequently results in impaired chewing, swallowing, and speaking, alongside facial disfigurement, severely diminishing quality of life and placing a considerable health burden on both patients and society [3,4]. With a five-year survival rate of only 40%-50%, it constitutes a significant global health issue [5]. Between 1975 and 2016, HNC incidence exhibited fluctuating trends, characterized by a pattern of increase-decrease-increase-stagnation [6]. In 2018, 354,864 new cases of lip and oral cavity cancer, 129,079 nasopharyngeal cancer cases, and 177,422 laryngeal cancer cases were reported, representing 2.0%, 0.7%, and 1.0% of global cancer diagnoses, respectively. Corresponding deaths reached 177,384, 72,987, and 94,771, comprising 1.9%, 0.8%, and 1.0% of global cancer mortality [1]. In China, approximately 128,000 new HNC cases and 65,000 deaths occur annually [7]. Studies show a correlation between age and rising incidence and mortality of lip and oral cavity and pharyngeal cancers [8]. According to China’s seventh national population census, 190 million individuals are aged 65 or older, accounting for 13.5% of the population [5]. Declining birth rates further complicate cancer control efforts [9], adding pressure to an already burdened healthcare system and necessitating greater focus on HNC epidemiological trends [6].

Recent demographic changes in China are expected to contribute to a rising incidence of HNC [7]. The COVID-19 pandemic in 2020 and 2021 posed unique challenges, compelling head and neck surgeons to adapt their diagnostic and treatment strategies [10]. However, the impact of China’s COVID-19-related public health measures on HNC burden remains unclear, with further data required for quantification.

Most previous research has concentrated on specific risk factors or subsites of HNC [5], leaving a gap in a comprehensive analysis of current and future trends in overall HNC burden. Additionally, no decomposition analysis of HNC incidence and mortality by anatomical site has been conducted in China.

The Global Burden of Disease (GBD) database, a widely used tool for quantifying morbidity, mortality, and Disability-Adjusted Life Years (DALYs) in China, facilitates the assessment of disease burden disparities and informs public health policies [11]. The recent GBD 2021 data release, including updated statistics for 2020 and 2021, presents an opportunity to reassess the demographic impact and HNC burden in China during the COVID-19 pandemic [12].

This study has three primary objectives. First, we aim to conduct a comprehensive numerical assessment and trend analysis of the disease burden of HNC in China over the past two decades. Second, our goal is to predict the potential trajectory of the disease burden over the next 25 years. Third, we seek to provide critical insights for governments and health policymakers in China and globally, supporting the development of targeted prevention and control strategies, optimizing policy decisions, and ensuring effective resource allocation.

## Materials and methods

### Data source

Metrics related to the disease burden of HNC were sourced from the GBD 2021 database (https://ghdx.healthdata.org/gbdresultstool, accessed September 1, 2024), provided by the Institute for Health Metrics and Evaluation (IHME) at the University of Washington, USA. In summary, the location selected was “China,” with “Lip and oral cavity cancer,” “Nasopharynx cancer,” and “Larynx cancer” chosen as the causes, and “incidence,” “death,” and “DALYs” as the measures. All estimates were reported with a 95% uncertainty interval (95% UI). To analyze the relationship between incidence, mortality, and DALYs by age, the population was divided into 20 age groups: < 5, 5–9, 10–14, 15–19, 20–24, 25–29, 30–34, 35–39, 40–44, 45–49, 50–54, 55–59, 60–64, 65–69, 70–74, 75–79, 80–84, 85–89, 90–94, and 95 + years. A comprehensive description of the general methods used in GBD 2021 is available on the official GBD Research website (https://ghdx.healthdata.org/gbd-2021). Additionally, this article does not include any studies involving human participants or animals conducted by the authors.

### Statistical analysis

The age-standardized rate (ASR), estimated average percentage change (EAPC), and the levels of all-age incidence, mortality, and DALYs were calculated to assess the burden of HNC subsites.

$ASR=\frac{\sum_{i=1}^{A}a_{i}w_{i}}{\sum_{i=1}^{A}w_{i}}\times 100,100$

In this context, $a_{i}$ denotes the age-specific rate for the ith age group, w represents the number of individuals (or the weight) within the same ith age group from the reference standard population, and A indicates the total number of age groups. The EAPC is calculated based on the formula y = a + bx + e, where y refers to ln (ASR) and x represents the calendar year [13]. EAPC is then derived using the formula 100 × (exp(b) – 1), and the 95% confidence interval (CI) can be obtained from the linear regression model. If the EAPC is greater than zero, the age-standardized indicator demonstrates an upward trend. Conversely, if EAPC is less than zero, the indicator shows a downward trend. A value of zero for the EAPC indicates that the indicator remains constant.

Subsequently, age-period-cohort (APC) analysis was performed to assess the influence of age, period, and cohort on the previously mentioned disease metrics [14]. This analysis employed a log-linear model incorporating additive effects of the three components—age, period, and birth cohort—represented by the following equation:

$\log\lambda =\alpha_{a}+\beta_{p}+\gamma_{c}$

In this context, α represents the age effect, β denotes the period effect, and γ signifies the cohort effect. The primary indicators of interest included: (1) local drifts, reflecting annual percentage changes in age-specific rates; (2) longitudinal age curves, showing the rate variation across age groups; (3) period rate ratios (RR), indicating the rate for a specific period relative to a reference period; and (4) cohort RRs, representing the ratio of each cohort’s rate compared to the reference cohort.

To analyze the factors driving changes in incidence and mortality numbers by cause, a decomposition analysis was conducted comparing 2021–2019. The observed changes in incidence and mortality were broken down into three components: changes due to population aging, changes in population structure by age or gender, and changes in the ASR. The net change in incidence and mortality reflects the combined effect of these three factors. This decomposition method follows the approach developed by the GBD 2015 Mortality and Causes of Death Collaborator [15].

The Autoregressive Integrated Moving Average (ARIMA) model is a powerful tool for analyzing cancer burden data, as it effectively captures trends, seasonal fluctuations, and random variations within time series. It is composed of two sub-models: the Autoregressive (AR) model, which accounts for the influence of past values, and the Moving Average (MA) model, which captures the effect of past errors [16]. Additionally, the integration step removes non-stationarity, a common challenge in time series data, thereby enabling more accurate forecasting [17]. This makes ARIMA particularly suitable for predicting the age-standardized incidence rate (ASIR) and age-standardized mortality rate (ASMR) of HNC from 2021 to 2046, where cancer patterns are complex and variable over time. All data analyses were performed using Excel 2019, R software (4.2.0, Free Software Foundation), and Prism (Version 10.1.1 (270), November 21, 2023).

## Results

### Changes in the worldwide ranking of ASIR and ASMR in China from 2000 to 2021

**Fig 1:**
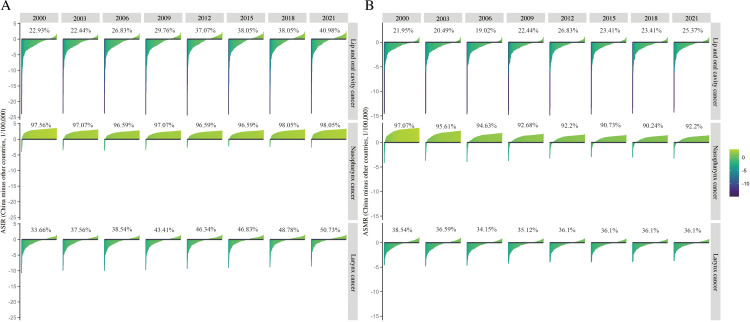
Global ranking of head and neck cancer subsite age-standardized incidence (ASIR) and mortality rates (ASMR) in China, 2000–2021. (A) ASIR of head and neck cancer subsites. (B) ASMR of head and neck cancer subsites.

The ASIR trends for 204 countries and regions from 2000 to 2021 reveal a gradual increase in the ASIR ranking of lip and oral cavity cancer in China, which ranked higher than 40.98% of countries worldwide by 2021 (Fig 1A). Nasopharyngeal cancer maintained a persistently high ASIR rank, exceeding 98.05% of countries in 2021, while laryngeal cancer’s ASIR rose steadily, surpassing 50.73% of global countries and regions during the same period (Fig 1A).

In terms of the ASMR, the global rankings from 2000 to 2021 show that the ASMR for lip and oral cavity cancer in China exceeded 25.37% of countries worldwide in 2021 (Fig 1B). Nasopharyngeal cancer ranked above 92.20% globally in ASMR, while laryngeal cancer ranked above 36.10% in 2021 (Fig 1B).

### Burden and trends of head and neck cancer in China from 2000 to 2021

In 2021, China estimated 161,190 new HNC cases and 75,000 related deaths. From 2000 to 2021, the ASIR for lip and oral cavity cancer in China increased by 51.4%, with an EAPC of 1.75% (95% CI: 1.52, 1.99), and the number of new cases rose by 188%, reaching 56,360 (95% UI: 45,180–69,680) in 2021 (Table 1). During the same period, the ASMR for lip and oral cavity cancer decreased by 0.9%, with an EAPC of -0.09 (95% CI: -0.25, 0.06) (Table 2). The ASMR for females exhibited a declining trend (EAPC: -1.8, 95% CI: -1.99, -1.6), whereas males showed an increasing trend (EAPC: 0.57, 95% CI: 0.39, 0.75). The number of deaths from lip and oral cavity cancer in 2021 was 23,880 (95% UI: 18,970, 29,680). DALYs associated with this cancer also showed a downward trend, with an EAPC of -0.19 (95% CI: -0.36, -0.01), while DALYs for females declined (EAPC: -1.95, 95% CI: -2.16, -1.73) and increased for males (EAPC: 0.47, 95% CI: 0.28, 0.66) (S1 Table).

**Table 2:** The deaths and ASMR of head and neck cancer in 2000 and 2021 and their temporal trends from 2000 to 2021.

Characteristics	2000		2021		2000-2021
	Numbers (thousands)	ASMR per 100,000	Numbers (thousands)	ASMR per 100,000	EAPC (%, 95%CI)
**Lip and oral cavity cancer**					
Both	11.94 (10.73,13.37)	1.16 (1.04,1.29)	23.88 (18.97,29.68)	1.15 (0.92,1.42)	-0.09 (-0.25,0.06)
Female	3.49 (3.07,4.02)	0.66 (0.58,0.77)	5.42 (4.26,6.72)	0.5 (0.4,0.62)	-1.8 (-1.99,-1.6)
Male	8.45 (7.47,9.62)	1.74 (1.54,1.96)	18.47 (13.78,23.84)	1.91 (1.45,2.45)	0.57 (0.39,0.75)
**Nasopharynx cancer**					
Both	36.19 (32.74,40.04)	3.13 (2.83,3.46)	31.32 (25.47,38.38)	1.51 (1.23,1.84)	-3.85 (-4.17,-3.54)
Female	10.63 (9.25,12.33)	1.85 (1.61,2.15)	7.92 (6.04,10.21)	0.74 (0.57,0.96)	-4.76 (-5.1,-4.42)
Male	25.56 (22.4,28.51)	4.49 (3.94,5.01)	23.4 (17.89,29.73)	2.32 (1.79,2.93)	-3.49 (-3.8,-3.18)
**Larynx cancer**					
Both	13.87 (12.14,15.66)	1.33 (1.16,1.49)	19.8 (15.58,25.02)	0.94 (0.74,1.17)	-1.74 (-1.83,-1.65)
Female	2.38 (1.63,2.85)	0.45 (0.3,0.54)	3.34 (1.98,4.6)	0.3 (0.18,0.42)	-2.13 (-2.23,-2.03)
Male	11.49 (10.03,13.14)	2.36 (2.07,2.69)	16.46 (12.22,21.22)	1.68 (1.27,2.13)	-1.68 (-1.77,-1.58)

Nasopharyngeal cancer’s ASIR decreased by 11.9% from 2000 to 2021, with an EAPC of -1.5% (95% CI: -1.93, -1.07) (Table 1), although the number of new cases rose by 37.6%, reaching 65,930 (95% UI: 53,270, 81,430) in 2021. During the same period, the ASMR for nasopharyngeal cancer declined by 51.8%, with an EAPC of -3.85 (95% CI: -4.17, -3.54) (Table 2), and deaths in 2021 were estimated at 31,320 (95% UI: 25,470, 39,440). DALYs for nasopharyngeal cancer followed a downward trend, with an EAPC of -3.92 (95% CI: -4.26, -3.57) (S1 Table).

**Table 1:** The incidence numbers and ASIR of head and neck cancer in 2000 and 2021 and their temporal trends from 2000 to 2021.

Characteristics	2000		2021		2000—2021
	Numbers (thousands)	ASIR per 100,000	Numbers (thousands)	ASIR per 100,000	EAPC (%, 95%CI)
**Lip and oral cavity cancer**					
Both	19.57 (17.59,21.78)	1.77 (1.59,1.98)	56.36 (45.18,69.8)	2.68 (2.15,3.3)	1.75 (1.52,1.99)
Female	6.41 (5.64,7.36)	1.14 (1,1.31)	14.71 (11.41,18.44)	1.38 (1.07,1.73)	0.3 (0.08,0.52)
Male	13.15 (11.61,15.02)	2.49 (2.2,2.82)	41.65 (31.15,54.22)	4.13 (3.12,5.32)	2.39 (2.13,2.65)
**Nasopharynx cancer**					
Both	47.92 (43.31,53.44)	3.88 (3.51,4.32)	65.93 (53.27,81.43)	3.42 (2.77,4.23)	-1.5 (-1.93,-1.07)
Female	14.46 (12.4,16.87)	2.36 (2.03,2.76)	16.06 (12.09,21.45)	1.69 (1.27,2.26)	-2.42 (-2.81,-2.03)
Male	33.46 (29.19,37.38)	5.44 (4.77,6.08)	49.87 (37.88,63.92)	5.16 (3.94,6.58)	-1.14 (-1.58,-0.71)
**Larynx cancer**					
Both	18.19 (15.8,20.51)	1.66 (1.45,1.87)	38.9 (30.37,49.49)	1.79 (1.4,2.26)	0.04 (-0.11,0.2)
Female	3.03 (2.07,3.63)	0.55 (0.38,0.66)	6.39 (3.87,8.78)	0.58 (0.35,0.79)	-0.27 (-0.41,-0.14)
Male	15.16 (13.16,17.29)	2.89 (2.53,3.3)	32.51 (24.07,42.39)	3.12 (2.34,4.04)	0.08 (-0.08,0.24)

For laryngeal cancer, the ASIR increased by 7.8% from 2000 to 2021, with an EAPC of 0.04 (95% CI: -0.11, 0.2) (Table 1). While the ASIR for females showed a decline (EAPC: -0.27, 95% CI: -0.41, -0.14), it increased for males (EAPC: 0.08, 95% CI: -0.08, 0.24). Incidence numbers rose by 113.9% during the period, reaching 38,900 new cases (95% UI: 30,370, 49,490) in 2021. The ASMR for laryngeal cancer decreased by 29.3%, with an EAPC of -1.74 (95% CI: -1.83, -1.65) (Table 2), while deaths were estimated at 19,800 (95% UI: 15,580, 25,020) in 2021. The DALYs for laryngeal cancer also declined, with an EAPC of -1.91 (95% CI: -2.01, -1.81) (S1 Table).

### Changes in the incidence and mortality of HNC by age and sex in China in 2021

**Fig 2:**
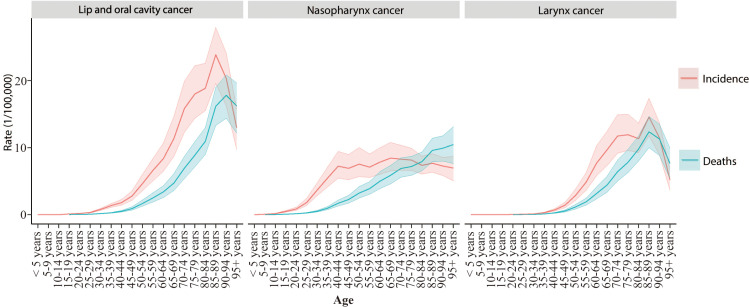
Incidence and mortality of head and neck cancer subsites by age in China, 2021.

Incidence and mortality rates of HNC by age subgroup were analyzed for 2021 (Fig 2). The incidence of lip and oral cavity cancer increases progressively with age, peaking between 80–94 years, followed by a decline. Mortality trends for lip and oral cavity cancer closely mirrored the incidence pattern. The incidence of nasopharyngeal cancer begins to rise between ages 20–39 and stabilizes after age 40, while mortality from nasopharyngeal cancer continues to increase with age. For laryngeal cancer, both incidence and mortality rates increase with age, peaking between 85–89 years, before declining. The DALYs for lip and oral cavity, nasopharynx, and larynx cancers also increase with age, peaking at ages 75–94, 55–74, and 70–89, respectively (S1 Fig).

**Fig 3:**
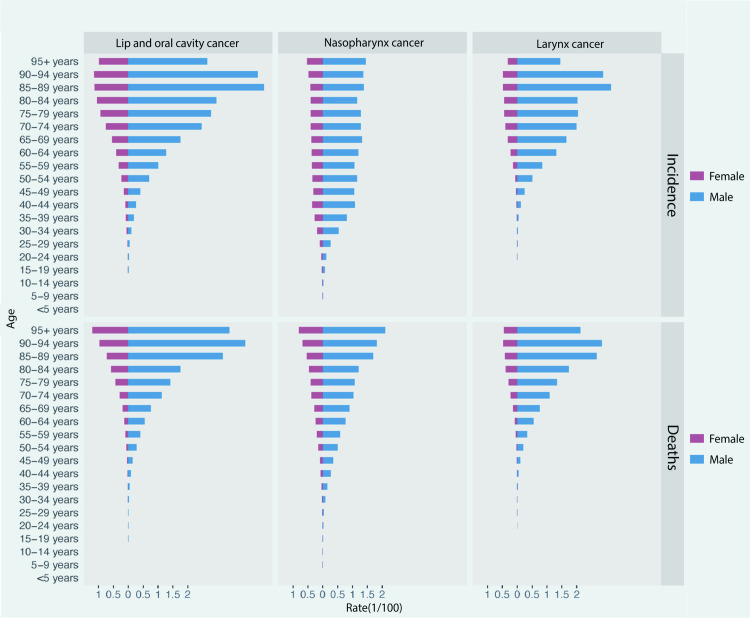
Incidence and mortality of head and neck cancer subsites by gender in China, 2021.

Gender differences in the incidence, mortality, and DALYs for HNC by age subgroup in 2021 are illustrated in Fig 3 and S2 Fig. In all age subgroups, men exhibited higher incidence, mortality, and DALYs for lip and oral cavity, nasopharyngeal, and laryngeal cancers compared to women.

### Age-period-cohort analysis of the incidence of head and neck cancer

**Fig 4:**
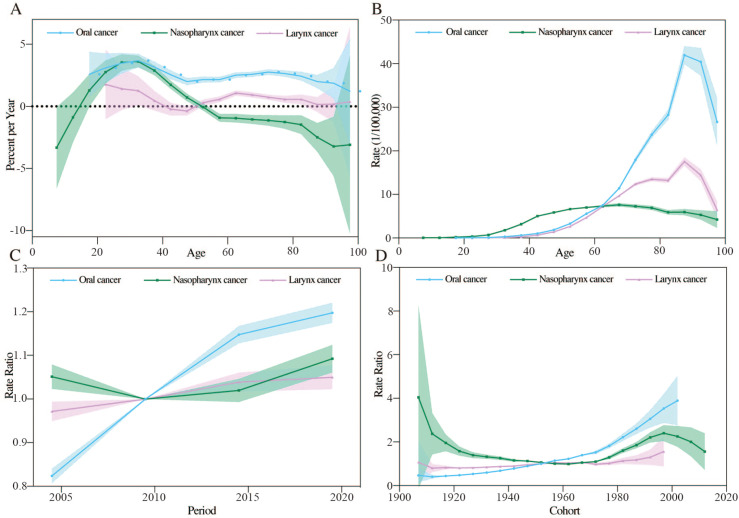
Age-period-cohort analysis of head and neck cancer incidence, 2000–2021. (A) Local drifts by age group. (B) Fitted longitudinal age curves of incidence. (C) Relative risk for each time period, using 2007–2011 as a reference. (D) Relative risk for each cohort, with the 1950–1954 cohort as a reference.

An APC model was employed to assess the effects of age, period, and cohort on HNC incidence between 2000 and 2021 (Fig 4). Local drift values showed positive trends for lip and oral cavity cancer across all age groups, nasopharyngeal cancer in individuals aged 20–50, and laryngeal cancer in those over 50 (Fig 4A). Regarding the age effect (Fig 4B), the incidence of lip and oral cavity cancer begins to rise around age 40, peaking at around 90 years. Nasopharyngeal cancer incidence increases gradually after age 20, with a peak between 40 and 60 years. Laryngeal cancer incidence similarly rises after 40, peaking around age 90. For the period effect (Fig 4C), the period RRs (relative to 2007–2011) for lip and oral cavity and laryngeal cancer steadily increased throughout the study period, while the RRs for nasopharyngeal cancer initially decreased, followed by a marked rise after 2010. The cohort effect revealed increasing cohort RRs (relative to 1950–1954) for lip and oral cavity and laryngeal cancers, with nasopharyngeal cancer peaking between 1995–1999 (Fig 4D).

### Decomposition analysis of incidence and deaths of HNC in China

**Fig 5:**
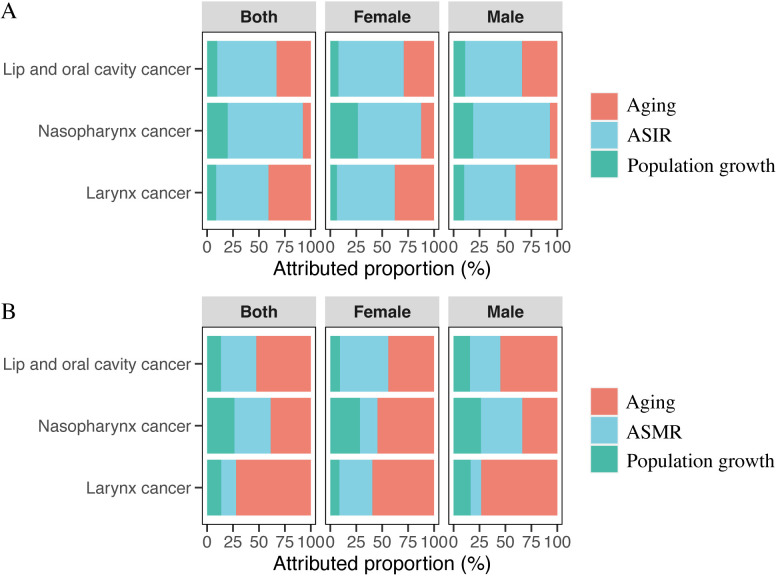
Decomposition analysis of head and neck cancer subsite incidence and mortality in China, 2019–2021. (A) Incidence of head and neck cancer subsites. (B) Mortality of head and neck cancer subsites.

Key factors driving changes in incidence numbers include population growth, aging, and ASIR (Fig 5A). Compared to 2019, the 2021 increase in lip and oral cavity, nasopharynx, and laryngeal cancer incidence was primarily driven by ASIR changes, accounting for 57.14%, 72.45%, and 50.41% respectively. Aging was the secondary driver for lip and oral cavity (33.05%) and laryngeal cancers (40.87%), whereas nasopharyngeal cancer was more influenced by population growth (19.83%).

For changes in the number of deaths, the primary contributors were population growth, aging, and ASMR (Fig 5B). Compared to 2019, the rise in deaths from lip and oral cavity, nasopharynx, and laryngeal cancers in 2021 was mainly driven by aging, contributing 52.61%, 38.76%, and 72.09%, respectively. In particular, nasopharyngeal cancer deaths in males were primarily driven by ASMR (39.85%). Secondary drivers were ASMR for lip and oral cavity (34.05%), nasopharyngeal (34.87%), and laryngeal cancers (14.34%). For nasopharyngeal cancer in males, aging was the secondary factor, contributing 33.75%.

### ASIR and ASMR of HNC in China projected for the next 25 years

The optimized model choices along with AIC, BIC, and AICC are presented in S2 Table. The observed and fitted values show good consistency. The Ljung-Box test confirms that the residuals of the model are white noise, with Q-values and P-values detailed in S2 Table. Except for laryngeal cancer in females, the residuals for all other cases exhibit a normal distribution based on the Q-Q plot, ACF plot, and PACF plot tests (S3 Fig).

**Fig 6:**
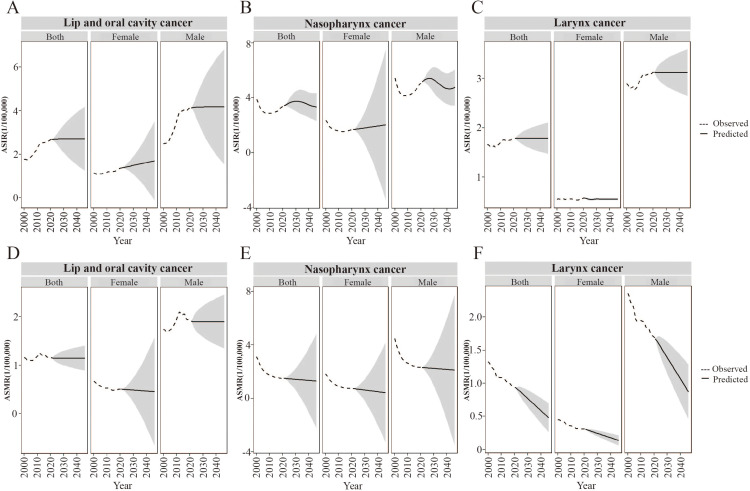
Projection of age-standardized incidence (ASIR) and mortality rates (ASMR) for head and neck cancer subsites in China, 2022–2046. (A) Projected ASIR for lip and oral cavity cancer. (B) Projected ASIR for nasopharynx cancer. (C) Projected ASIR for larynx cancer. (D) Projected ASMR for lip and oral cavity cancer. (E) Projected ASMR for nasopharynx cancer. (F) Projected ASMR for larynx cancer.

The ARIMA model was used to forecast the trends of ASIR and ASMR for HNC subsites in China from 2022 to 2046 (Fig 6). The ASIR for oral cancer is projected to rise in both females and males (Fig 6A). The ASIR for nasopharyngeal cancer is expected to increase and then decrease in females, with a peak occurring in 2026 in males (Fig 6B). The ASIR for laryngeal cancer is predicted to remain stable for both genders (Fig 6C).

Regarding ASMR, lip and oral cavity cancer is expected to remain stable in males but slightly decline in females (Fig 6D). The ASMR for nasopharyngeal cancer is projected to decrease slightly for both genders, while the ASMR for laryngeal cancer is predicted to significantly decline in both females and males (Fig 6E and 6F).

## Discussion

The newly updated GBD 2021 database provided the foundation for a comprehensive analysis of HNC trends in China over the past two decades. Findings reveal that China’s global ranking in the incidence of lip, oral cavity, and laryngeal cancers has risen, while nasopharyngeal cancer continues to hold a prominent position worldwide. These trends highlight the pressing need for increased focus on HNC in China, a deeper understanding of its contributing factors, and necessary policy adjustments.

Over the past 20 years, the ASIR for oral cavity and laryngeal cancers has demonstrated a continuous upward trajectory, likely driven by greater exposure to key risk factors. Smoking, a significant risk factor for HNC, accounts for 30% of the global smoking population in China [18,19]. Tobacco sales in the country increased steadily from 2004, peaking in 2014, with annual cigarette sales stabilizing at approximately 2.4 trillion [20]. Alcohol consumption is another critical risk factor [21], with the Global Alcohol and Health Report 2018 indicating a rise in per capita alcohol consumption in China from 4.1L in 2005 to 7.2L in 2016 [22]. Interestingly, the ASIR of nasopharyngeal cancer in both men and women has shown a downward trend, potentially due to improved public awareness of chemical protection. Formaldehyde exposure, a known risk factor for nasopharyngeal cancer, has been declining in homes, schools, and offices across China since 2002 [23].

Despite the rising ASIR for HNC, ASMR has decreased for all three malignancies, likely due to advancements in early diagnosis and treatment. The discovery of novel biomarkers, such as circulating tumor DNA (ctDNA), microRNA, and protein markers in saliva and blood, has significantly enhanced early detection of HNC [24–26]. For instance, Epstein-Barr virus (EBV) DNA has been identified as a reliable biomarker for nasopharyngeal carcinoma screening, especially in high-risk populations in southern China [27]. In terms of treatment, targeted therapies have become pivotal, particularly for patients with advanced or recurrent HNC [28]. Monoclonal antibodies like cetuximab, which targets the epidermal growth factor receptor (EGFR), have shown efficacy in improving survival when used in combination with radiation therapy or chemotherapy [29]. Furthermore, recent clinical trials exploring immune checkpoint inhibitors, such as pembrolizumab and nivolumab, have yielded promising results in treating HNC [30]. Notably, the ASMR for oral cancer has increased in men, potentially due to their higher exposure to risk factors like smoking and alcohol abuse.

In 2021, a detailed analysis of HNC incidence and mortality in China identified significant age- and sex-related trends. The increase in HNC incidence and mortality with age is likely due to cumulative exposure to risk factors such as tobacco use, alcohol consumption, and human papillomavirus infections, all of which exhibit prolonged latency periods [31]. Older adults are particularly vulnerable, as accumulated genetic mutations and a weakening immune system heighten cancer susceptibility, with lip, oral cavity, and laryngeal cancers peaking in elderly populations. Additionally, men are more frequently exposed to occupational carcinogens than women, contributing significantly to the development of laryngeal and oral cancers [32]. Therefore, promoting health education and implementing early screening for older men will be critical in reducing HNC incidence in China.

The results of the APC model indicate that local drifts in lip, oral cavity, and nasopharyngeal cancers were more pronounced in individuals aged 20–40, aligning with prior research [33,34]. The fast-paced, pressure-filled lifestyles resulting from socio-economic development have led younger populations to engage in smoking and alcohol consumption as coping mechanisms [35]. These findings underscore the need for future cancer prevention strategies, particularly those focused on mitigating risk factors among younger individuals. Moreover, the APC cohort reveals an increase in lip, oral cavity, and nasopharyngeal cancer incidence among individuals born after 1975, likely driven by increased occupational hazard exposure as industrialization progresses. To reduce the disease burden associated with these risk factors, implementing effective interventions is essential. Targeted education on smoking cessation and alcohol reduction for high-risk groups, particularly men, will raise awareness of disease prevention. Consequently, refining alcohol control measures and continuously enforcing smoking control policies should be top public health priorities. Additionally, stronger protections against occupational hazards are equally vital. This includes developing and enforcing protective measures to minimize exposure to harmful materials and equipment, maintaining occupational health records, and conducting regular health check-ups for workers.

To further explore the changes in HNC morbidity and mortality in China during the COVID-19 pandemic, a decomposition analysis was performed to compare the HNC population in 2021 with that in 2019. The findings indicate that ASIR and aging were the primary factors influencing the number of cases and deaths. This trend may be attributed to the significant diversion of healthcare resources toward pandemic management, potentially impacting the diagnosis and treatment of other diseases. Nevertheless, healthcare systems maintained substantial focus on screening and diagnosing malignancies, including HNC. Despite resource constraints, healthcare institutions optimized processes and improved efficiency, ensuring the timely screening and diagnosis of HNC, contributing to increased early case detection and, consequently, higher ASIR. Furthermore, with China’s aging population, the number of elderly individuals has grown. As a high-risk group during the pandemic, older adults received special attention, with enhanced health monitoring and diagnostic services. However, due to inherent physiological vulnerabilities, they continue to represent a significant contributor to HNC mortality.

Predictive models for the next 25 years project an increase in HNC incidence in China, accompanied by a decrease in mortality, suggesting prolonged survival among patients. Consequently, improving patients’ quality of life will be essential. Clinicians must optimize treatment approaches to preserve patient function and promote functional reconstruction [36]. Additionally, healthcare professionals should focus on strengthening postoperative psychological counseling and rehabilitation to alleviate depression, manage pain, and support functional recovery [37]. Simultaneously, further exploration of HNC risk factors is necessary, with the development of personalized risk assessments aimed at encouraging behavioral change, facilitating the early detection of precancerous lesions, and promoting timely treatment to reduce the societal burden of HNC.

Our study has several limitations. First, the limited availability of epidemiological data on HNC in China, coupled with significant heterogeneity across studies, may introduce bias between GBD estimates and actual data. Although GBD incorporates a broad spectrum of published and unpublished data, the data on HNC remains insufficient, as many of the registries used may not fully represent all HNC cases in China. Second, HNC encompasses diverse subtypes, such as highly differentiated squamous cell carcinoma and sarcoma, yet the lack of comprehensive data from GBD impedes detailed subgroup analyses based on clinical characteristics. Finally, due to insufficient granularity in the available data, it was not possible to stratify the burden of laryngeal cancer by province, economic development level, or ethnicity within China.

Future research should focus on several key areas to better understand and address the burden of HNC, particularly regarding the specific mechanisms of established factors such as smoking, alcohol consumption, and HPV infection, as well as emerging risks like e-cigarettes and environmental pollution. The development and application of molecular biomarkers, including circulating tumor DNA and microRNAs, hold significant potential for early diagnosis and personalized treatment. Epidemiological data on HNC in China remain insufficient, especially concerning regional, socioeconomic, and ethnic variations, necessitating enhanced data collection and analysis for more accurate burden estimates. Optimizing prevention and early screening strategies, particularly for high-risk groups, should be prioritized, alongside large-scale clinical trials. Furthermore, research into innovative treatment approaches, especially in combination therapies involving immunotherapy and targeted therapies, is crucial to improving patient outcomes. Lastly, the long-term impact of the COVID-19 pandemic on HNC diagnosis, treatment, and healthcare delivery should be thoroughly evaluated to understand its lasting effects.

## Conclusion

The incidence of HNC in China has followed a upward trajectory over the past 20 years and is expected to continue rising in the next 25 years, particularly among older men. Of particular concern is the increasing incidence of HNC in younger adults. Despite this, the projected decline in ASMR suggests that advancements in healthcare will continue to alleviate the mortality burden. These findings underscore the necessity of reinforcing public health interventions and maintaining a strong emphasis on early detection and innovative treatment options to tackle the escalating challenge of HNC in China.

## Supporting information

S1 Fig Disability-adjusted life years (DALYs) rate of head and neck cancer subsites by age in China, 2021. (TIF)

S2 Fig Disability-adjusted life years (DALYs) rate of head and neck cancer subsites by gender in China, 2021. (TIF)

S3 Fig The ARIMA model’s Q-Q plot, ACF plot, and PACF plot were utilized to test whether the residuals are normally distributed for the prediction of Age-Standardized Incidence Rate (ASIR) and Age-Standardized Mortality Rate (ASMR) for Head and Neck Cancer over the next 25 years in China. (A) ASIR for lip and oral cavity cancer. (B) ASIR for nasopharynx cancer. (C) ASIR for larynx cancer. (D) ASMR for lip and oral cavity cancer. (E) ASMR for nasopharynx cancer. (F) ASMR for larynx cancer.
(TIF)

S1 Table The numbers of DALYs and Age-Standardized DALYs rate of head and neck cancer in 2000 and 2021 and their temporal trends from 2000 to 2021. (DOCX)

S2 Table ARIMA model parameters and their corresponding AIC, BIC, AICC, Q, and P for prediction of ASIR and ASMR for Head and Neck Cancer for the next 25 years in China. (DOCX)

## Abbreviations

HNC: Head and Neck Cancer
GBD: Global Burden of Disease
APC: Age-Period-Cohort
ASIR: age-standardized incidence rate
ASMR: age-standardized incidence
DALYs: Disability-Adjusted Life Years
