# Supplementary figures and images for: Trends, levels, and projections of Head and Neck Cancer in China between 2000 and 2021: Findings from the Global Burden of Disease 2021
Source: PLoS One. 2025 May 2;20(5):e0322533. doi: 10.1371/journal.pone.0322533 (PMC12047823; doi:10.1371/journal.pone.0322533)

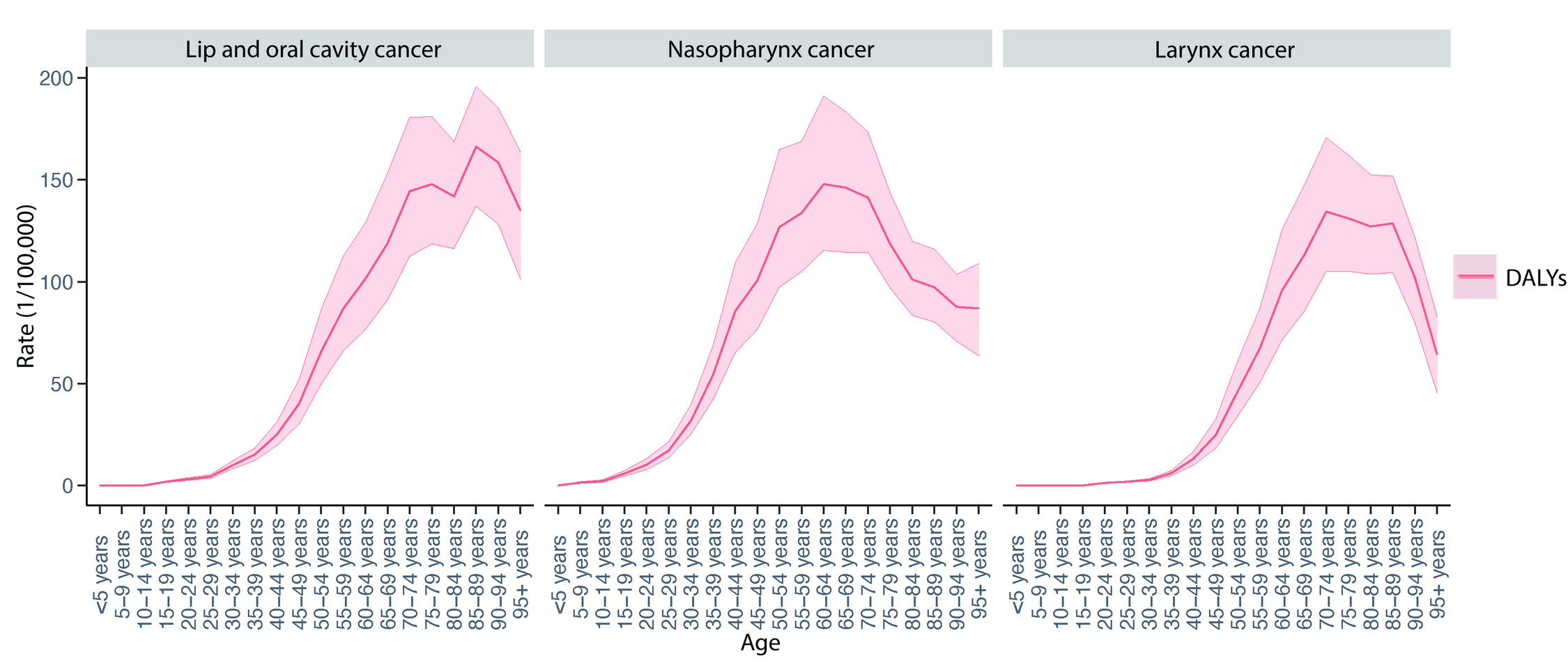

Supplement: S1 Fig — (TIF) [file pone.0322533.s001.tif]

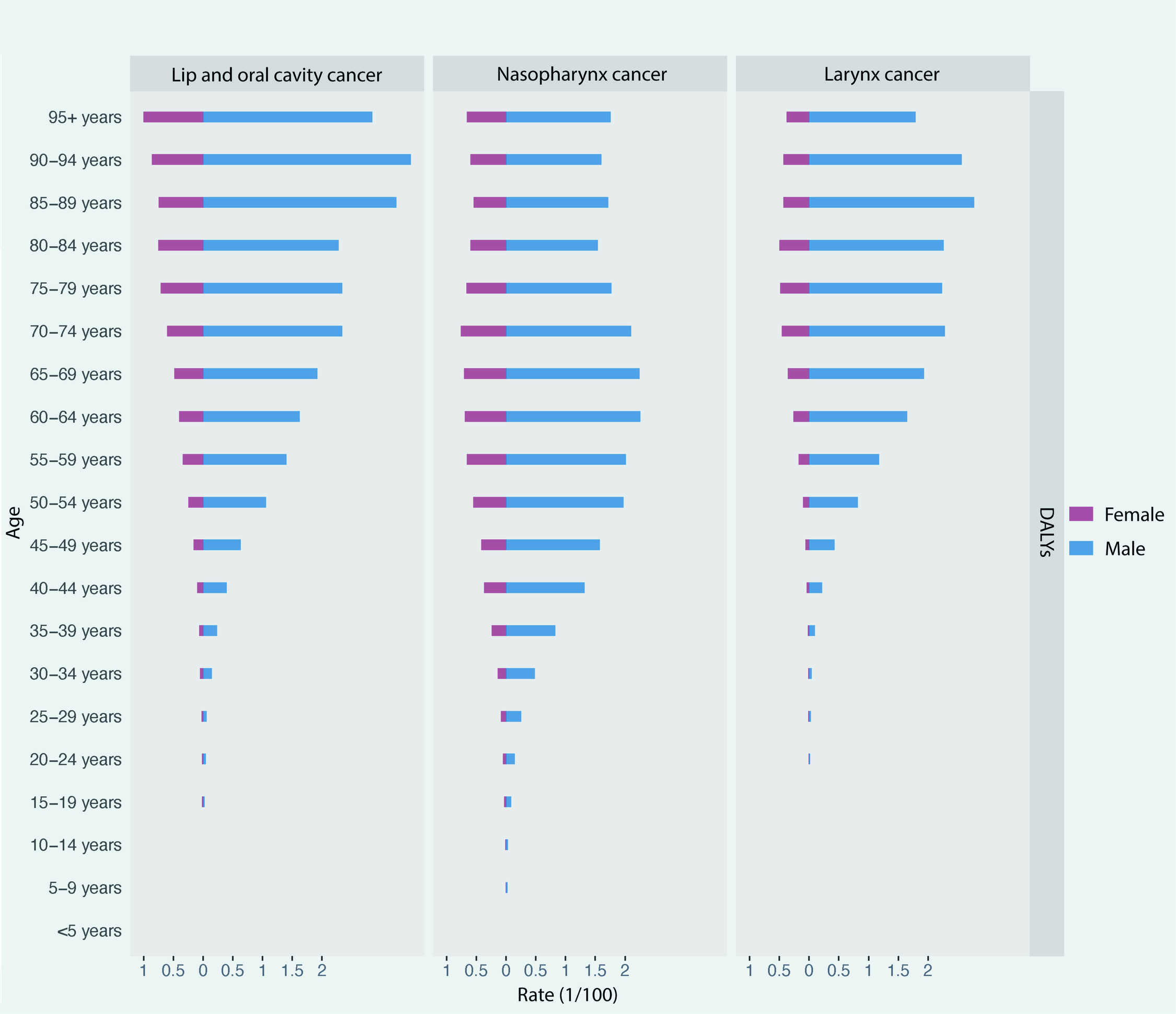

Supplement: S2 Fig — (TIF) [file pone.0322533.s002.tif]

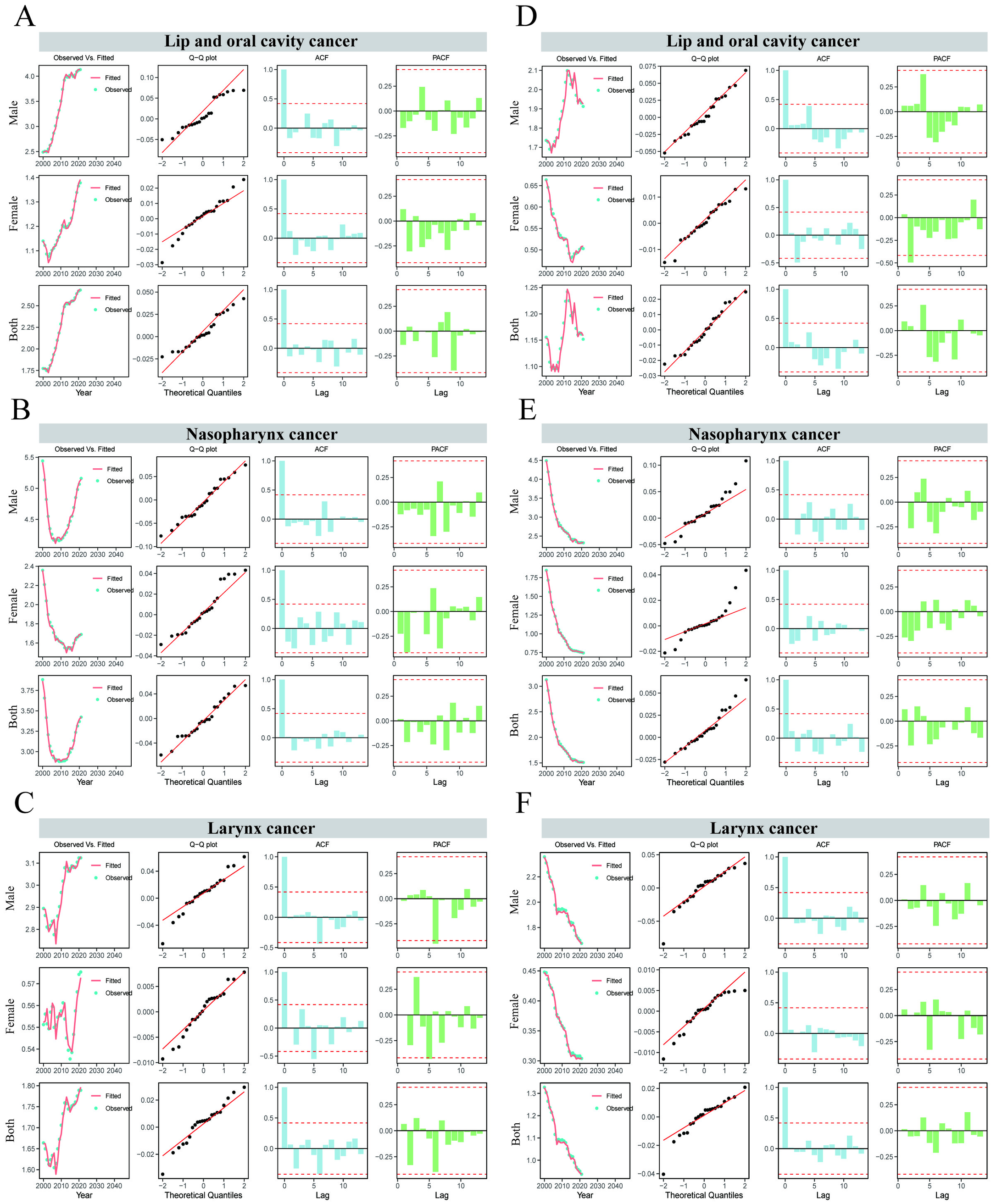

Supplement: S3 Fig — (A) ASIR for lip and oral cavity cancer. (B) ASIR for nasopharynx cancer. (C) ASIR for larynx cancer. (D) ASMR for lip and oral cavity cancer. (E) ASMR for nasopharynx cancer. (F) ASMR for larynx cancer. (TIF) [file pone.0322533.s003.tif]
