# Supplementary material for: Trends, levels, and projections of Head and Neck Cancer in China between 2000 and 2021: Findings from the Global Burden of Disease 2021
Source: PLoS One. 2025 May 2;20(5):e0322533. doi: 10.1371/journal.pone.0322533 (PMC12047823; doi:10.1371/journal.pone.0322533)
Supplement: S1 Table — (DOCX) [file pone.0322533.s004.docx]

S1 Table. The numbers of DALYs and Age-Standardized DALYs rate of head and neck cancer in 2000 and 2021 and their temporal trends from 2000 to 2021.

| Characteristics | 2000 | | 2021 | | 2000—2021 |
| --- | --- | --- | --- | --- | --- |
|  | Numbers (thousands) | ASR per 100,000 | Numbers (thousands) | ASR per 100,000 | EAPC  (%, 95% CI) |
| Lip and oral cavity cancer |  |  |  |  |  |
| Both | 341.62 (307.61,381.15) | 29.38 (26.4,32.86) | 618.02 (487.51,777.18) | 29.2 (23.18,36.49) | -0.19 (-0.36,-0.01) |
| Female | 97.13 (85.31,111.69) | 16.73 (14.64,19.25) | 132.73 (102.62,167.24) | 12.45 (9.61,15.71) | -1.95 (-2.16,-1.73) |
| Male | 244.49 (215.6,279.82) | 42.62 (37.7,48.42) | 485.29 (359.8,634.36) | 47.06 (35.1,60.94) | 0.47 (0.28,0.66) |
| Nasopharynx cancer |  |  |  |  |  |
| Both | 1260.63 (1140.69,1404.06) | 99.67 (90.17,110.88) | 982.66 (797.64,1210.38) | 48.67 (39.59,59.48) | -3.92 (-4.26,-3.57) |
| Female | 362.61 (312.83,421.57) | 58.43 (50.5,67.92) | 233.84 (176.24,305.91) | 23.05 (17.4,30.28) | -4.93 (-5.28,-4.57) |
| Male | 898.02 (786.33,1002.38) | 140.3 (122.6,156.35) | 748.82 (566.35,952.74) | 74.35 (56.63,94.46) | -3.5 (-3.84,-3.16) |
| Larynx cancer |  |  |  |  |  |
| Both | 377.25 (327.9,426.32) | 32.91 (28.65,37.28) | 493.85 (382.57,626.01) | 22.73 (17.67,28.65) | -1.91 (-2.01,-1.81) |
| Female | 61.13 (39.75,72.69) | 10.73 (7.15,12.8) | 77.08 (46.25,106.17) | 7.01 (4.19,9.66) | -2.32 (-2.42,-2.22) |
| Male | 316.12 (273.48,362.5) | 56.2 (48.88,64.22) | 416.77 (304.82,544.63) | 39.58 (29.17,51.44) | -1.8 (-1.91,-1.69) |
