# Supplementary material for: Trends, levels, and projections of Head and Neck Cancer in China between 2000 and 2021: Findings from the Global Burden of Disease 2021
Source: PLoS One. 2025 May 2;20(5):e0322533. doi: 10.1371/journal.pone.0322533 (PMC12047823; doi:10.1371/journal.pone.0322533)
Supplement: S2 Table — (DOCX) [file pone.0322533.s005.docx]

S2 Table. ARIMA model parameters and their corresponding AIC, BIC, AICC, Q, and P for prediction of ASIR and ASMR for Head and Neck Cancer for the next 25 years in China.

|  |  | Parameters | AIC | BIC | AICC | Q | P |
| --- | --- | --- | --- | --- | --- | --- | --- |
| ASIR | Lip and oral cavity cancer |  |  |  |  |  |  |
|  | Both | ARIMA (1,1,1) | -97.321 | -94.187 | -95.909 | 0.974 | 0.807 |
|  | Female | ARIMA (0,2,0) | -116.559 | -115.563 | -116.337 | 3.145 | 0.534 |
|  | Male | ARIMA (1,1,1) | -66.960 | -63.827 | -65.549 | 3.501 | 0.321 |
|  | Nasopharynx cancer |  |  |  |  |  |  |
|  | Both | ARIMA (2,0,0) | -74.268 | -69.904 | -71.915 | 1.380 | 0.710 |
|  | Female | ARIMA (0,2,1) | -89.744 | -87.752 | -89.038 | 5.576 | 0.134 |
|  | Male | ARIMA (2,0,1) | -59.763 | -54.308 | -56.013 | 3.653 | 0.301 |
|  | Larynx cancer |  |  |  |  |  |  |
|  | Both | ARIMA (1,1,0) | -111.604 | -109.515 | -110.938 | 3.565 | 0.312 |
|  | Female | ARIMA (2,0,0) | -165.486 | -161.122 | -163.133 | 14.905 | 0.002 |
|  | Male | ARIMA (0,1,1) | -83.665 | -81.576 | 82.998 | 0.304 | 0.959 |
| ASMR | Lip and oral cavity cancer |  |  |  |  |  |  |
|  | Both | ARIMA (0,1,1) | -115.305 | -113.216 | -114.639 | 2.279 | 0.516 |
|  | Female | ARIMA (0,2,0) | -135.791 | -134.795 | -135.569 | 7.364 | 0.118 |
|  | Male | ARIMA (0,1,1) | -82.000 | -79.911 | -81.333 | 4.774 | 0.189 |
|  | Nasopharynx cancer |  |  |  |  |  |  |
|  | Both | ARIMA (0,2,0) | -89.586 | -88.590 | -89.363 | 2.444 | 0.655 |
|  | Female | ARIMA (0,2,1) | -107.029 | -105.038 | -106.323 | 4.205 | 0.240 |
|  | Male | ARIMA (0,2,0) | -71.135 | -70.140 | -70.913 | 4.530 | 0.339 |
|  | Larynx cancer |  |  |  |  |  |  |
|  | Both | ARIMA (0,1,1) | -116.098 | -112.964 | 114.686 | 0.642 | 0.887 |
|  | Female | ARIMA (0,1,1) | -161.071 | -157.938 | -159.659 | 0.641 | 0.887 |
|  | Male | ARIMA (0,1,1) | -85.885 | -82.752 | -84.474 | 0.979 | 0.806 |
